# The Relationship Between Body Mass Index and Cervical High-Risk HPV Positivity in Women: A Single-Center Study

**DOI:** 10.3390/microorganisms14030555

**Published:** 2026-02-28

**Authors:** Cemal Çiçek, Mehmet Alican Sapmaz, Ayfer Bakır, Elif Tuğçe Güner, Murat Aral

**Affiliations:** 1 Department of Medical Microbiology, University of Health Science Ankara Etlik City Hospital, 06170 Ankara, Türkiye; dr.ayfer.bakir@gmail.com (A.B.); eliftugce06md@gmail.com (E.T.G.); aralmurat@hotmail.com (M.A.); 2 Department of Obstetrics and Gynecology, University of Health Science Ankara Etlik City Hospital, 06170 Ankara, Türkiye; dr.alicansapmaz@hotmail.com

**Keywords:** Human papillomavirus, body mass index, obesity, genotype, PCR, cytology

## Abstract

Background: Human papillomavirus (HPV) is the primary etiological agent of cervical cancer. Although obesity has been proposed as a factor influencing HPV acquisition and disease course through immune and metabolic mechanisms, its role remains controversial. This study aimed to evaluate the association between body mass index (BMI) and high-risk (HR)-HPV infection, including genotype distribution, infection type, and cytological findings. Methods: This cross-sectional study included women aged 21 years and older who underwent cervical sampling between August and November 2025. Participants were classified as non-obese (BMI <30 kg/m^2^) or obese (BMI ≥30 kg/m^2^). HR-HPV genotypes were detected using a multiplex real-time PCR method, and cytological evaluation was performed according to the Bethesda Cervical Cytology Reporting System. Results: Among 518 women, the overall HR-HPV positivity rate was 13.5%. No significant difference in HR-HPV positivity was observed between obese (11.6%) and non-obese (14.2%) women (OR = 0.79; 95% CI: 0.44–1.44; *p* = 0.452). After age adjustment, obesity was not identified as an independent risk factor for HR-HPV infection. BMI was not associated with HPV genotype distribution, infection type, or cytological findings (all *p* > 0.05). HPV-68 was the most frequently detected genotype. Conclusions: BMI was not independently associated with HR-HPV infection or related clinical and cytological features. These findings suggest that HPV infection is primarily influenced by viral characteristics and host immune response, while BMI appears to play a limited role. Further multicenter prospective studies are needed to clarify the impact of obesity on HPV infection.

## 1. Introduction

Human papillomavirus (HPV) is one of the most common sexually transmitted infection agents and is an important global health problem, playing a role in approximately 5% of all cancers worldwide [1]. HPV is a small, non-enveloped, double-stranded DNA virus that infects epithelial tissues, belongs to the *Papillomaviridae* family, and more than 200 genotypes have been identified to date [2,3]. It is estimated that more than 80% of sexually active women will experience at least one HPV infection by the age of 45 [1,4]. More than 90% of HPV infections are asymptomatic and resolve spontaneously within two years; however, persistent infections with high-risk (HR)-HPV types may progress to precancerous lesions and invasive cancers [5]. Human papillomavirus (HPV) has been classified as a Group 1 human carcinogen and has been causally linked to six cancer types, including cervical, anal, vulvar, vaginal, penile, and oropharyngeal cancers [2]. Cervical cancer, in particular, is largely associated with HPV and remains the fourth most common cancer among women worldwide, with a substantial proportion of cases attributed to HPV infection [4]. HR genotypes, especially HPV-16 and HPV-18, play a primary role in cervical carcinogenesis [6].

Understanding the host-related factors that influence the epidemiology and progression of HPV infection is critical for the development of effective prevention and control strategies [7]. In this context, body mass index (BMI) has recently attracted increasing attention as a potential modifier of HPV acquisition, persistence, and disease progression [8]. Several studies have reported that women with higher BMI values exhibit increased HPV positivity rates and a higher risk of cervical cancer; however, the biological mechanisms underlying this association remain incompletely understood [9,10].

Obesity is thought to induce immune dysregulation, chronic low-grade inflammation, and metabolic disturbances, thereby increasing susceptibility to viral infections [11,12,13]. Accordingly, metabolic syndrome has been associated with persistent cervical HPV infection and may impair immune clearance through inflammatory cytokines, adipokines, and oxidative stress pathways [14,15,16,17,18]. In addition, insulin, insulin-like growth factors, and estrogen have been suggested to contribute to cervical carcinogenesis by acting synergistically with HPV oncogenes [15,18]. Despite these mechanistic hypotheses, epidemiological findings regarding the relationship between BMI and HPV infection or cervical cancer risk remain inconsistent [19,20,21,22,23,24,25].

Although women with higher BMI are hypothesized to be more susceptible to HPV infection and to have a reduced capacity for viral clearance, the association between BMI and HPV infection remains controversial [9,20]. These conflicting findings indicate that the relationship between BMI and HR-HPV infection has not yet been clearly established. Therefore, clarifying this relationship is essential both for identifying epidemiological risk groups and for improving targeted prevention and surveillance strategies.

Accordingly, the present study aimed to investigate the association between BMI and HR-HPV infection, and to evaluate whether BMI constitutes a potential risk factor for HPV positivity, genotype distribution, infection type (single or multiple), and cytological abnormalities.

## 2. Materials and Methods

### 2.1. Study Design

This study was designed as a cross-sectional observational, single-center study based on prospectively collected data and was conducted in collaboration with the Medical Microbiology Clinic and the Department of Obstetrics and Gynecology at Etlik City Hospital.

Participants were recruited from women who attended the Gynecology and Obstetrics outpatient clinics between 1 August 2025, and 30 November 2025, and from whom cervical samples were obtained as part of routine clinical evaluation and HPV testing. All participants provided written informed consent prior to inclusion.

Women aged 21 years and older who were scheduled for cervical sampling, had complete BMI data, and voluntarily agreed to participate were eligible for inclusion. The lower age limit was selected to allow evaluation of the association between BMI and HPV infection across the reproductive age spectrum, independent of age-specific screening recommendations. This approach was chosen to investigate epidemiological relationships rather than to assess screening performance.

Exclusion criteria included pregnancy; postpartum or breastfeeding status; a history of malignancy with ongoing chemotherapy or radiotherapy; impaired consciousness; intensive care unit admission; or legal incapacity to provide informed consent.

Sample size was calculated using G*Power software version 3.1.9.7 (Heinrich Heine University, Düsseldorf, Germany) to detect a difference in HR-HPV positivity between the BMI < 30 kg/m^2^ and BMI ≥ 30 kg/m^2^ groups. Allowing for potential data loss, a total of 518 women were included in the study.

BMI was calculated as weight in kilograms divided by height in meters squared (kg/m^2^). Participants were classified using two approaches. In the four-category classification, BMI values were defined as underweight (<18.5 kg/m^2^), normal weight (18.5–<25 kg/m^2^), overweight (25–<30 kg/m^2^), and obese (≥30 kg/m^2^). For statistical comparison, a second dichotomous classification was applied, defining BMI < 30 kg/m^2^ as non-obese and BMI ≥ 30 kg/m^2^ as obese. Both classifications were used according to the requirements of the analyses.

### 2.2. Sample Collection

Cervical smear samples were collected by a gynecology specialist using a sterile Dacron swab and transferred into a vNAT Transfer Tube (Cat. No: BS-NA-513m-100; Bioeksen R&D Technologies, Istanbul, Türkiye). The collected samples were transported to the laboratory under appropriate biosafety and transport conditions and stored at +4 °C until processing on the same day. This system allows nucleic acid stabilization, enabling direct PCR analysis without the need for an additional nucleic acid isolation step.

### 2.3. Molecular Analysis

The High-Risk Human Papillomavirus qPCR Panel kit (Cat. No: BS-HRHPV-L25/BS-HRHPV-L100, Bioeksen R&D Technologies, Istanbul, Türkiye) was used for the analysis of nucleic acids obtained from cervical smear samples.

A test based on the principle of multiplex real-time polymerase chain reaction (multiplex real-time PCR) was used for the simultaneous detection of HR-HPV genotypes. This test targeted the E1–E7 and L1 regions of the HPV genome. The kit aimed to detect 14 HR-HPV genotypes (HPV 16, 18, 31, 33, 35, 39, 45, 51, 52, 56, 58, 59, 66, and 68) with high sensitivity. All procedures were carried out according to the manufacturer’s recommendations. The sensitivity and specificity of the Bioeksen HPV qPCR Panel, as reported by the manufacturer, were 99.4% and 99.6%, respectively.

In the analyses, positive control, negative control, and internal control provided by the manufacturer were used. To achieve a total reaction volume of 20 µL, 10 µL of DNA sample was added to each reaction. Amplifications were carried out using the Magnetic Induction Cycler (Mic)-PCR system (Bio Molecular Systems, Upper Coomera, Gold Coast, QLD, Australia).

The analysis of the results was performed automatically using Sigmoida software (Version 8.6 REV.56; Bioeksen R&D Technologies, Istanbul, Türkiye). Samples were considered positive when a Ct value ≤30 was obtained together with a valid amplification curve according to the manufacturer’s baseline and threshold criteria. Samples with internal control failure were retested.

### 2.4. Cytological Evaluation

The concurrently obtained cervical cytology results of the patients were retrieved from the Hospital Information Management System. Cytological evaluations were performed in the hospital pathology unit according to the routine diagnostic workflow based on the Bethesda Cervical Cytology Reporting System. Cytology results were classified as negative for intraepithelial lesion or malignancy (NILM), atypical squamous cells of undetermined significance (ASC-US), atypical squamous cells cannot exclude high-grade squamous intraepithelial lesion (ASC-H), low-grade squamous intraepithelial lesion (LSIL), and high-grade squamous intraepithelial lesion (HSIL). For statistical analysis, normal cytology findings and benign or reactive cellular changes were combined into a single group (normal/benign) because they do not represent a clinical risk for malignancy. Samples were considered unsatisfactory when they showed insufficient cellular material for cytological evaluation or failed internal control amplification during molecular analysis, in accordance with routine laboratory quality criteria.

### 2.5. Statistical Analysis

All statistical analyses were performed using IBM SPSS Statistics version 26.0 (IBM Corp., Armonk, NY, USA). The distribution of continuous variables was assessed using the Kolmogorov–Smirnov test. Variables showing normal distribution were summarized as mean ± standard deviation (SD), while non-normally distributed variables were presented as median and interquartile range (IQR). Categorical variables were expressed as number (*n*) and percentage (%).

Comparisons of categorical variables between groups were performed using Pearson’s chi-square test or Fisher’s exact test when appropriate. The Mann–Whitney U test was used to compare continuous variables between two groups. HPV positivity rates were evaluated according to BMI categories (four-group and binary classifications) and age groups. Differences in HPV positivity and genotype distributions among cytology groups were analyzed using the chi-square test. To evaluate the independent association between HPV positivity and BMI categories (<30 and ≥30 kg/m^2^), multivariable logistic regression analysis was performed after adjustment for age. Results were expressed as odds ratios (ORs) with 95% confidence intervals (CIs). A two-tailed *p* value of <0.05 was considered statistically significant for all analyses.

## 3. Results

### 3.1. Demographic and Clinical Characteristics of the Participants

The median age of the 518 women included in the study was 40.0 years (IQR: 34.0–47.0; range: 21–77 years). It was observed that the vast majority of participants (75.1%) were in the 30–49 age range. According to BMI distribution, 37.8% of the women were classified as normal weight, 34.7% as overweight, and 26.6% as obese, while only 0.8% were underweight.

When cervical cytology results were examined, it was determined that the vast majority of women (90.0%) had normal cytology findings, whereas abnormal cytology results were observed at low frequencies. Detailed data on the participants’ demographic and clinical characteristics, as well as the distribution of BMI and smear cytology, are presented in Supplementary Table S1.

### 3.2. HPV Positivity Status, Distribution According to Genotypes and Age Groups

HPV positivity was detected in 13.5% (*n* = 70) of the total 518 cases. The median ages of HPV-positive and HPV-negative cases were 40 years (range: 28–63; IQR: 33.0–48.0) and 40 years (range: 21–77; IQR: 35.0–47.0), respectively, and no statistically significant difference was observed between the two groups (*p* = 0.653).

HPV positivity rates across age groups ranged from 11.6% to 14.9%, and no statistically significant difference was found among age groups (*p* = 0.849).

In the majority of HPV infected cases, single genotype positivity was observed (10.2%), while multiple genotype positivity was detected at a lower rate (3.3%), with no statistically significant difference between these patterns (*p* = 0.810).

Among HPV-positive cases, the most frequently detected genotype was HPV-68 (2.3%), followed by HPV-16 (2.1%), HPV-31 (2.1%), and HPV-45 (1.8%). Other genotypes (HPV-18, HPV-33, HPV-35, HPV-39, HPV-51, HPV-52, HPV-56, HPV-58, HPV-59, and HPV-66) were detected at lower rates (each <1.5%). No statistically significant difference in genotype distribution was observed across age groups (all *p* > 0.05) (Supplementary Table S2).

### 3.3. HPV Positivity Distribution Across Cytology Groups According to BMI Levels

The distribution of HPV positivity across cytological groups according to BMI categories is presented in Supplementary Table S3. In the BMI < 30 kg/m^2^ group, 89.5% of cases had normal cytology, while 9.2% were ASC-US, 1.1% LSIL, and 0.3% ASC-H. Similarly, in the BMI ≥ 30 kg/m^2^ group, 91.3% of cases showed normal cytology and 8.7% had ASC-US, with no LSIL or ASC-H cases observed. No statistically significant difference was found in cytological distribution between BMI groups (*p* = 0.783).

HPV positivity rates tended to increase with cytological severity; however, no statistically significant association was observed between HR-HPV positivity and cytology groups when stratified by BMI (*p* = 0.441).

**Figure 1:**
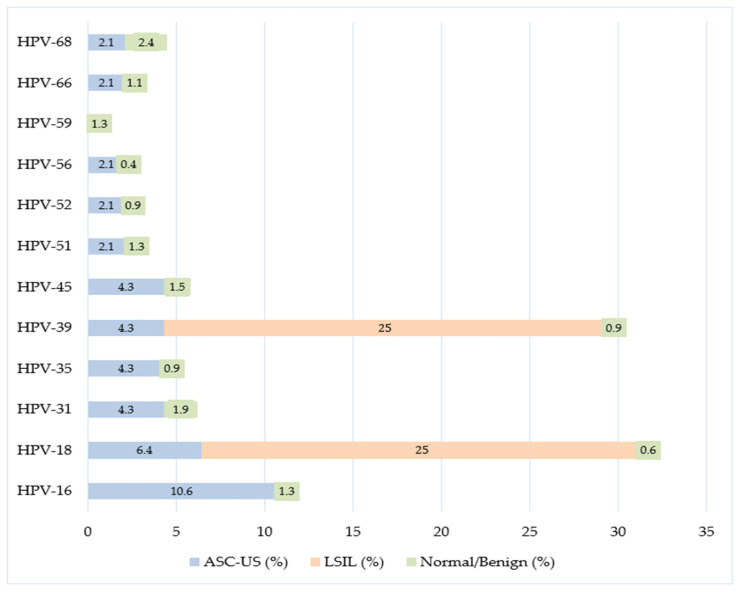
Positivity rates of HR-HPV types according to cytology groups.

Figure 1 shows the distribution of HR-HPV genotypes according to cytology groups. In the ASC-US group, HPV-16 (10.6%) and HPV-18 (6.4%) were the most frequently detected genotypes. In the LSIL group, only HPV-18 (25%) and HPV-39 (25%) were observed. In women with normal or benign cytology, HPV-68 was the most frequently detected genotype (2.4%); however, HPV-16, HPV-18, and HPV-39 were more prevalent in abnormal cytology groups, as shown in Figure 1.

**Figure 2:**
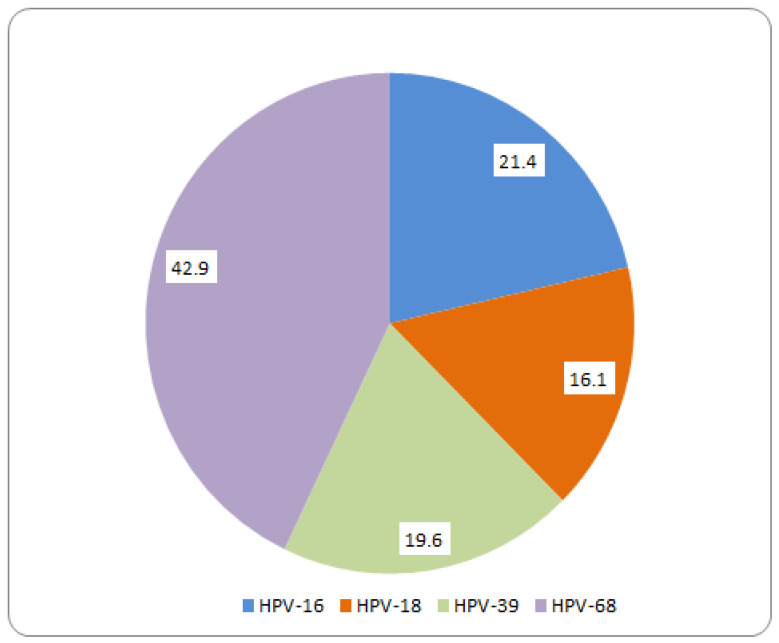
Distribution of HR-HPV genotypes in normal cytology.

Figure 2 further illustrates the distribution of HR-HPV genotypes among women with normal or benign cytology, highlighting the predominance of HPV-68 within this subgroup.

### 3.4. Distribution of Body Mass Index and HR-HPV Positivity

The median BMI value in HPV-negative individuals was 26.36 (IQR: 23.51–30.75), whereas in HPV-positive individuals it was 25.67 (IQR: 23.53–29.31), with no statistically significant difference between the two groups (*p* = 0.326).

According to the four-group BMI classification, no statistically significant difference was observed in HPV positivity rates (*p* = 0.716). While no HPV positivity was detected in the BMI < 18.5 kg/m^2^ group, positivity rates in the remaining BMI categories ranged from 11.6% to 14.8%.

Similarly, according to the binary classification, no statistically significant difference was observed in HR-HPV positivity rates between the BMI < 30 and BMI ≥ 30 groups (*p* = 0.441) (Supplementary Table S4).

**Figure 3:**
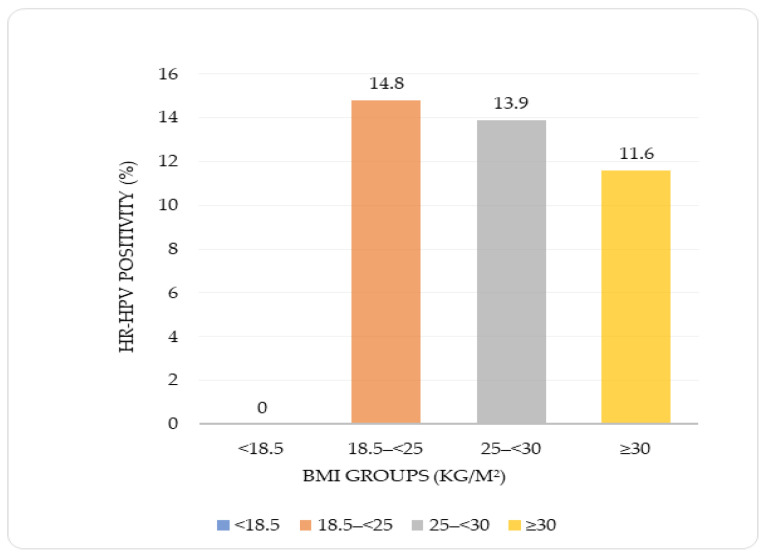
HR-HPV positivity rates according to BMI groups.

Figure 3 illustrates HR-HPV positivity rates across BMI categories, showing no statistically significant differences between BMI groups.

Table 1 presents the types of HR-HPV infection (single and multiple) and genotype distributions according to BMI groups (<30 and ≥30 kg/m^2^). In both BMI groups, single infections were the predominant infection pattern (BMI < 30: 11.3%; BMI ≥ 30: 7.2%), and no statistically significant difference was observed between the groups with respect to infection type (*p* = 0.306).

Among HR-HPV genotypes, HPV-68 exhibited the highest overall prevalence (2.3%). The distribution of the remaining genotypes was comparable between the two BMI groups, and no statistically significant differences were detected for any HPV genotype according to BMI category (all *p* > 0.05).

### 3.5. Distribution of the Relationship Between Body Mass Index and HPV Positivity According to Age Groups

HPV positivity was evaluated according to BMI categories (four-group classification) and age groups, and no statistically significant association was observed (*p* = 0.837). HPV positivity rates were 14.8% in normal-weight individuals (18.5–<25 kg/m^2^), 13.9% in the overweight group (25–<30 kg/m^2^), and 11.6% in the obese group (≥30 kg/m^2^). No HPV positivity was detected in underweight individuals (<18.5 kg/m^2^).

Using the binary BMI classification, HR-HPV positivity rates were 14.2% in the BMI < 30 kg/m^2^ group and 11.6% in the BMI ≥ 30 kg/m^2^ group, with no statistically significant difference between the groups (*p* = 0.441) (Table 2).

### 3.6. Risk Analysis of HR-HPV Infection in Obese Women

Table 3 presents the risk analysis related to HPV positivity in non-obese (<30 kg/m^2^) and obese (≥30 kg/m^2^) women. The overall HR-HPV positivity rate was 11.6% in the obese group and 14.2% in the non-obese group, with no statistically significant difference between the groups (OR = 0.79; 95% CI: 0.44–1.44; *p* = 0.452).

In subgroup analyses according to age categories, no statistically significant association was identified between obesity and HR-HPV positivity in any age group (all *p* > 0.05). Similarly, genotype-based analyses revealed no significant association between obesity and any specific HR-HPV genotype (all *p* > 0.05).

Multivariable logistic regression analysis was performed to evaluate the effects of age and BMI on HPV positivity. No statistically significant association was observed between age and HR-HPV positivity (OR = 0.996; 95% CI: 0.969–1.024; *p* = 0.785). Although HR-HPV positivity was higher in individuals with BMI ≥ 30 kg/m^2^ compared to those with BMI < 30 kg/m^2^, this increase was not statistically significant (OR = 1.25; 95% CI: 0.686–2.278; *p* = 0.466) (Table 4). The explanatory power of the model was low (Nagelkerke R^2^ = 0.002), indicating that age and BMI contributed only minimally to HR-HPV positivity.

## 4. Discussion

In this single-center cross-sectional study, the association between BMI and HR-HPV infection was evaluated in a screening population of women. The main finding of the study is that BMI was not significantly associated with HR-HPV positivity, genotype distribution, infection type, or cytological abnormalities. These results remained consistent in both unadjusted analyses and age-adjusted multivariable logistic regression models, indicating that obesity is not an independent risk factor for HR-HPV infection in this population.

The relationship between obesity and HPV infection has been reported inconsistently in the literature. While some studies suggest that obesity may increase the risk of HPV positivity and cervical neoplasia [7,9,23], others have demonstrated no association or even a lower prevalence of HR-HPV infection in obese individuals [20,21,24]. These discrepancies may be attributed to differences in study design, population characteristics, HPV detection methods, adjustment for confounding variables, and regional epidemiological patterns.

Obesity is known to be associated with immune dysregulation, chronic low-grade inflammation, and metabolic alterations, which theoretically may impair viral clearance and promote persistent HPV infection [14,15,16]. However, several studies have reported that these biological effects do not consistently trans-late into increased HPV prevalence at the clinical or epidemiological level [20,25]. The current results support that obesity has a limited direct impact on the acquisition of HR-HPV infection.

Importantly, null findings such as those reported in the present study are epidemiologically valuable, as they help to reduce publication bias and provide a more balanced understanding of HPV infection dynamics [26,27]. HPV infection is a multifactorial process influenced by viral, host, behavioral, and environmental factors [28,29]. Therefore, the absence of a significant association between BMI and HR-HPV positivity suggests that BMI alone is insufficient to explain individual susceptibility to HPV infection [20,22,24,30].

The overall HR-HPV positivity rate in this study was 13.5%, which is slightly higher than the global average but consistent with previously reported prevalence ranges from Türkiye [28]. Variations in HPV prevalence across regions are well recognized and may reflect differences in sexual behavior, screening practices, and diagnostic methodologies [31].

Age is another important determinant of HPV epidemiology. Although several studies have reported a bimodal age distribution of HPV prevalence, with peaks in younger and older age groups [32,33,34]. No significant age-related differences were observed in the present study. This may be explained by the cross-sectional design, sample distribution, and population-specific demographic and behavioral characteristics.

The most frequently detected genotype in this study was HPV-68, highlighting the importance of regional and population-specific genotype variability. While HPV-16 and HPV-18 are globally dominant genotypes, geographical differences in genotype distribution are well documented [32,35]. Reports from regions such as Madagascar have similarly identified HPV-68 as a prevalent genotype [36]. In Türkiye, however, HPV-16, HPV-18, HPV-31, HPV-52, and HPV-58 are more commonly reported [37,38]. These findings indicate that HPV genotype distribution is shaped primarily by geographic, sociocultural, and viral ecological factors rather than by single host-related factors such as BMI.

No significant association was found between BMI and cytological abnormalities in this study. This supports previous reports suggesting that cytological progression is more strongly related to viral genotype, viral load, and host immune response than to obesity itself [39,40,41]. Although obesity may indirectly influence cervical carcinogenesis through chronic inflammation and metabolic dysregulation, its direct contribution to cytological progression remains unclear.

Technical difficulties during gynecological examination in obese women may also affect cytological sampling quality, potentially leading to under-detection of lesions [42,43]. Therefore, future studies incorporating histopathological confirmation and long-term follow-up are needed to clarify this relationship.

At the biological level, HPV-related carcinogenesis is driven by complex interactions involving metabolic reprogramming, adipokines, immune modulation, and vaginal microbiota alterations [42,43,44]. These multidimensional pathways support the notion that obesity affects HPV infection indirectly through complex biological networks rather than as a single dominant determinant.

The absence of significant associations between BMI, age, and HR-HPV positivity in this study is consistent with several previous population-based investigations [20,24,30]. However, due to the cross-sectional design, causal inferences cannot be made.

This study has several limitations. First, its single-center design may limit generalizability. Second, the cross-sectional nature precludes evaluation of HPV persistence, clearance, and lesion progression. Third, lifestyle and behavioral factors such as smoking, sexual behavior, contraceptive use, physical activity, and dietary habits were not comprehensively assessed and may have introduced residual confounding. Fourth, metabolic and inflammatory biomarkers were not included, preventing direct evaluation of biological mechanisms. Finally, the low frequency of abnormal cytology and multiple infections limited the statistical power of subgroup analyses. Additionally, the absence of HSIL cases in the present study precludes definitive conclusions regarding whether BMI constitutes a risk factor for HSIL.

Despite these limitations, the adequate sample size, standardized molecular testing, comprehensive genotype analysis, and age-adjusted statistical modeling strengthen the reliability of the findings.

## 5. Conclusions

Overall, the present study demonstrates that BMI is not an independent determinant of HR-HPV infection, genotype distribution, infection type, or cytological abnormalities. These findings support the concept that the natural history of HPV infection is primarily shaped by viral characteristics and host immune responses, while BMI plays a limited role.

## Figures and Tables

**Table 1:** Distribution of HR-HPV positivity and genotype profiles according to BMI groups (<30 and ≥30 kg/m^2^).

	BMI < 30 (*n*, %)	BMI ≥ 30 (*n*, %)	Total (*n*, %)	*p* Value
Infection types				
Single	43 (11.3)	10 (7.2)	53 (10.2)	0.306
Multiple	11 (2.9)	6 (4.3)	17 (3.3)	
HR-HPV types				
HPV-16	7 (1.8)	4 (2.9)	11 (2.1)	0.461
HPV-18	6 (1.6)	1 (0.7)	7 (1.4)	0.457
HPV-31	9 (2.4)	2 (1.4)	11 (2.1)	0.521
HPV-33	1 (0.3)	0 (0.0)	1 (0.2)	0.546
HPV-35	6 (1.6)	0 (0.0)	6 (1.2)	0.138
HPV-39	4 (1.1)	3 (2.2)	7 (1.4)	0.329
HPV-45	8 (2.1)	1 (0.7)	9 (1.7)	0.288
HPV-51	5 (1.3)	2 (1.4)	7 (1.4)	0.907
HPV-52	5 (1.3)	0 (0.0)	5 (1.0)	0.176
HPV-56	2 (0.5)	1 (0.7)	3 (0.6)	0.793
HPV-58	3 (0.8)	1 (0.7)	4 (0.8)	0.941
HPV-59	5 (1.3)	1 (0.7)	6 (1.2)	0.578
HPV-66	5 (1.3)	1 (0.7)	6 (1.2)	0.578
HPV-68	7 (1.8)	5 (3.6)	12 (2.3)	0.234

Abbreviations: BMI, body mass index; HR-HPV, high-risk human papillomavirus; *n*, number of case.

**Table 2:** Distribution of HR-HPV positivity according to BMI and age groups.

BMI Group (kg/m^2^)	21–29 yrs (N, %)	30–39 yrs (N, %)	40–49 yrs (N, %)	≥50 yrs (N, %)	Total (N, %)
Total (four-group)					
<18.5	0/1 (0.0)	0/2 (0.0)	0/1 (0.0)	0 (0.0)	0/4 (0.0)
18.5–<25	2/12 (16.7)	13/94 (13.8)	6/58 (10.3)	8/32 (25.0)	29/196 (14.8)
25–<30	2/11 (18.2)	13/72 (18.1)	8/59 (13.6)	2/38 (5.3)	25/180 (13.9)
≥30	0 (0.0)	5/49 (10.2)	6/54 (11.1)	5/31 (16.1)	16/138 (11.6)
*p* value	0.999	0.613	0.888	0.072	0.837
Total (two-group)					
<30	4/24 (16.7)	26/168 (15.5)	14/118 (11.9)	10/70 (14.3)	54/380 (14.2)
≥30	0/4 (0.0)	5/49 (10.2)	6/54 (11.1)	5/31 (16.1)	16 (11.6)
*p* value	0.999	0.487	0.999	0.810	0.441

Abbreviations: BMI, body mass index; yrs, years; N, number of cases.

**Table 3:** Risk analysis related to HR-HPV positivity in non-obese and obese individuals.

	Non-Obese (<30 kg/m^2^)		Obese (≥30 kg/m^2^)		Odds Ratio	95% CI	*p* Value
	HPV (−)	HPV (+)	HPV (−)	HPV (+)			
HR-HPV status	326	54	122	16	0.79	0.44–1.44	0.452
Age groups							
21–29	20	4	4	0	0.62	0.22–1.71	0.366
30–39	142	26	44	5	0.93	0.32–2.68	0.888
40–49	104	14	48	6	0.79	0.32–2.56	0.807
≥50	60	10	26	5	1.15	0.35–3.71	0.810
HR-HPV types							
HPV-16	323	8	132	4	1.53	0.46–5.52	0.465
HPV-18	374	2	137	1	1.53	0.05–48.4	0.846
HPV-31	371	9	137	2	0.16	0.03–1.84	0.146
HPV-33	379	6	138	0	0.16	0.01–2.84	0.196
HPV-35	394	1	138	0	0.91	0.06–22.55	0.956
HPV-39	376	6	135	3	0.99	0.46–2.26	0.993
HPV-45	372	8	137	3	0.53	0.21–1.57	0.344
HPV-51	375	5	138	2	0.59	0.12–2.45	0.479
HPV-52	375	5	136	2	1.13	0.21–5.75	0.884
HPV-56	378	3	137	0	0.48	0.05–4.53	0.518
HPV-58	377	5	137	1	0.27	0.03–2.37	0.243
HPV-59	375	5	135	1	0.27	0.03–2.54	0.259
HPV-66	375	5	135	1	0.55	0.06–4.73	0.585
HPV-68	373	7	133	5	1.85	0.58–5.91	0.284

Abbreviations: HR-HPV, human papillomavirus; BMI, body mass index; OR, odds ratio; CI, confidence interval.

**Table 4:** Multivariable logistic regression analysis of the effect of age and BMI on HR-HPV positivity.

Variable	B	S.E.	Wald	df	*p*	OR	95% CI
Age	−0.004	0.014	0.075	1	0.785	0.996	0.969–1.024
BMI ≥30	0.223	0.306	0.531	1	0.466	1.250	0.686–2.278

BMI was included in the model as a categorical variable, with BMI <30 kg/m^2^ as the reference group. Age was included as a continuous variable. B: regression coefficient; S.E.: standard error; Wald: Wald chi-square test statistic; df: degrees of freedom; OR: odds ratio; CI: confidence interval. ORs are presented with 95% confidence intervals. Statistical significance was defined as *p* < 0.05.

## Data Availability

The data presented in this study are available on request from the corresponding author due to ethical and privacy restrictions.

## Supplementary Materials

The following supporting information can be downloaded at https://www.mdpi.com/article/10.3390/microorganisms14030555/s1, Supplementary Table S1: Demographic and clinical characteristics of the study population; Supplementary Table S2: Distribution of HR-HPV infection status by age group; Supplementary Table S3: HR-HPV positivity according to cytology groups by BMI; Supplementary Table S4: Distribution of HR-HPV positivity according to BMI groups.

## Abbreviations

The following abbreviations are used in this manuscript:

ASC-H	Atypical squamous cells—cannot exclude high-grade squamous intraepithelial lesion
ASC-US	Atypical squamous cells of undetermined significance
BMI	Body mass index
CI	Confidence interval
Ct	Cycle threshold
DNA	Deoxyribonucleic acid
HPV	Human papillomavirus
HR-HPV	High-risk human papillomavirus
HSIL	High-grade squamous intraepithelial lesion
IQR	Interquartile range
LSIL	Low-grade squamous intraepithelial lesion
NILM	Negative for intraepithelial lesion or malignancy
OR	Odds ratio
PCR	Polymerase chain reaction
qPCR	Quantitative polymerase chain reaction
SD	Standard deviation
