# Supplementary material for: The Relationship Between Body Mass Index and Cervical High-Risk HPV Positivity in Women: A Single-Center Study
Source: Microorganisms. 2026 Feb 28;14(3):555. doi: 10.3390/microorganisms14030555 (PMC13028971; doi:10.3390/microorganisms14030555)
Supplement: Supplementary file 1 [file microorganisms-14-00555-s001.zip › Supplementary Table S1.pdf]

Supplementary Table S1. Demographic and clinical characteristics of the study population

Variable	Category	n	%
BMI (kg/m²)			
	< 18.5	4	0.8
	18.5 – < 25	196	37.8
	25 – < 30	180	34.7
	≥ 30	138	26.6
BMI group (kg/m²)			
	< 30	380	73.4
	≥ 30	138	26.6
Age group (years)			
	21 – 29	28	5.4
	30 – 39	217	41.9
	40 – 49	172	33.2
	≥ 50	101	19.5
Smear cytology			
	ASC-H	1	0.2
	ASC-US	47	9.1
	LSIL	4	0.8
	Normal	466	90.0

Abbreviations: BMI, body mass index; ASC-H, atypical squamous cells—cannot exclude HSIL; ASC-US, atypical squamous cells of undetermined significance; LSIL, low-grade squamous intraepithelial lesion. ndicates the number of participants in each category. Percentages are calculated based on the total study population (n = 518).
