# Supplementary material for: The Relationship Between Body Mass Index and Cervical High-Risk HPV Positivity in Women: A Single-Center Study
Source: Microorganisms. 2026 Feb 28;14(3):555. doi: 10.3390/microorganisms14030555 (PMC13028971; doi:10.3390/microorganisms14030555)
Supplement: Supplementary file 1 [file microorganisms-14-00555-s001.zip › Supplementary Table S2.pdf]

**Supplementary Table S2**. Distribution of HR-HPV infection status by age group

| Characteristics | Age groups (yrs) 21–29 (n, %) | 30–39 (n, %) | 40–49 (n, %) | ≥ 50 (n, %) | Total (n, %) | P value |
|---|---|---|---|---|---|---|
| HR-HPV status | | | | | | |
| Negative | 24 (85.7) | 186 (85.7) | 152 (88.4) | 86 (85.1) | 448 (86.5) | 0.849 |
| Positive | 4 (14.3) | 31 (14.3) | 20 (11.6) | 15 (14.9) | 70 (13.5) | |
| Infection type | | | | | | |
| Single | 2 (7.1) | 24 (11.1) | 16 (9.3) | 11 (10.9) | 53 (10.2) | 0.810 |
| Multiple | 2 (7.1) | 7 (3.2) | 4 (2.3) | 4 (4.0) | 17 (3.3) | |
| HR-HPV types | | | | | | |
| HPV-16 | 2 (7.1) | 6 (2.8) | 1 (0.6) | 2 (2.0) | 11 (2.1) | 0.122 |
| HPV-18 | 0 (0.0) | 2 (0.9) | 2 (1.2) | 3 (3.0) | 7 (1.4) | 0.438 |
| HPV-31 | 0 (0.0) | 8 (3.7) | 3 (1.7) | 0 (0.0) | 11 (2.1) | 0.141 |
| HPV-33 | 0 (0.0) | 0 (0.0) | 0 (0.0) | 1 (1.0) | 1 (0.2) | 0.247 |
| HPV-35 | 1 (3.6) | 1 (0.5) | 2 (1.2) | 2 (2.0) | 6 (1.2) | 0.401 |
| HPV-39 | 0 (0.0) | 2 (0.9) | 4 (2.3) | 1 (1.0) | 7 (1.4) | 0.571 |
| HPV-45 | 1 (3.6) | 3 (1.4) | 4 (2.3) | 1 (1.0) | 9 (1.8) | 0.708 |
| HPV-51 | 1 (3.6) | 4 (1.8) | 1 (0.6) | 1 (1.0) | 7 (1.4) | 0.514 |
| HPV-52 | 0 (0.0) | 5 (2.3) | 0 (0.0) | 0 (0.0) | 5 (1.0) | 0.072 |
| HPV-56 | 0 (0.0) | 0 (0.0) | 2 (1.2) | 1 (1.0) | 3 (0.6) | 0.433 |
| HPV-58 | 0 (0.0) | 1 (0.5) | 1 (0.6) | 2 (2.0) | 4 (0.8) | 0.476 |
| HPV-59 | 0 (0.0) | 4 (1.8) | 1 (0.6) | 1 (1.0) | 6 (1.2) | 0.628 |
| HPV-66 | 0 (0.0) | 2 (0.9) | 2 (1.2) | 2 (2.0) | 6 (1.2) | 0.794 |
| HPV-68 | 2 (7.1) | 5 (2.3) | 3 (1.7) | 2 (2.0) | 12 (2.3) | 0.364 |

Abbreviations: HR-HPV, high-risk human papillomavirus; yrs, years; n, number of case.
