# Supplementary material for: The Relationship Between Body Mass Index and Cervical High-Risk HPV Positivity in Women: A Single-Center Study
Source: Microorganisms. 2026 Feb 28;14(3):555. doi: 10.3390/microorganisms14030555 (PMC13028971; doi:10.3390/microorganisms14030555)
Supplement: Supplementary file 1 [file microorganisms-14-00555-s001.zip › Supplementary Table S3.pdf]

Supplementary Table S3. HR-HPV positivity according to cytology groups by BMI

Cytology	BMI <30<br>% (n/N)	BMI ≥30<br>% (n/N)	Total (N=518)<br>% (n/N)	p value
ASC-H	0 (0/1)	-	0 (0/1)	-
ASC-US	31.4 (11/35)	33.3 (4/12)	31.9 (15/47)	0.999
LSIL	25.0 (1/4)	-	25.0 (1/4)	-
Normal/Benign	12.4 (42/340)	9.5 (12/126)	11.6 (54/466)	0.397
Total	14.2 (54/380)	11.6 (16/138)	13.5 (70/518)	0.441

Abbreviations**:** BMI, body mass index; ASC-US, atypical squamous cells of undetermined significance; LSIL, low-grade squamous intraepithelial lesion; n, number of HPV positive cases; N, total number of cases within each cytology category.
