# Supplementary material for: The Relationship Between Body Mass Index and Cervical High-Risk HPV Positivity in Women: A Single-Center Study
Source: Microorganisms. 2026 Feb 28;14(3):555. doi: 10.3390/microorganisms14030555 (PMC13028971; doi:10.3390/microorganisms14030555)
Supplement: Supplementary file 1 [file microorganisms-14-00555-s001.zip › Supplementary Table S4.pdf]

Supplemantary Table S4. Distribution of HR-HPV positivity according to BMI groups

BMI Group (kg/m²)	HPV Negative (n, %)	HPV Positive (n, %)	Total (n)	p value
Four-group				
<18.5	4 (100.0)	0 (0.0)	4	
18.5 – <25	167 (85.2)	29 (14.8)	196	0.716
25 – <30	155 (86.1)	25 (13.9)	180	
≥ 30	122 (88.4)	16 (11.6)	138	
Two-group				
< 30	326 (85.8)	54 (14.2)	380	0.441
≥ 30	122 (88.4)	16 (11.6)	138	
Total	448 (86.5)	70 (13.5)	518	

Abbreviations: BMI, body mass index; HR-HPV, high-risk human papillomavirus; n, number of case.
